# Different Transcriptional Response to *Xanthomonas citri* subsp. *citri* between Kumquat and Sweet Orange with Contrasting Canker Tolerance

**DOI:** 10.1371/journal.pone.0041790

**Published:** 2012-07-26

**Authors:** Xing-Zheng Fu, Xiao-Qing Gong, Yue-Xin Zhang, Yin Wang, Ji-Hong Liu

**Affiliations:** 1 Key Laboratory of Horticultural Plant Biology (MOE), National Key Laboratory of Crop Genetic Improvement, College of Horticulture and Forestry Science, Huazhong Agricultural University, Wuhan, China; 2 Citrus Research Institute, Chinese Academy of Agricultural Sciences, Chongqing, China; United States Department of Agriculture, United States of America

## Abstract

Citrus canker disease caused by *Xanthomonas citri* subsp. *citri* (Xcc) is one of the most devastating biotic stresses affecting the citrus industry. Meiwa kumquat (*Fortunella crassifolia*) is canker-resistant, while Newhall navel orange (*Citrus sinensis* Osbeck) is canker-sensitive. To understand the molecular mechanisms underlying the differences in responses to Xcc, transcriptomic profiles of these two genotypes following Xcc attack were compared by using the Affymetrix citrus genome GeneChip. A total of 794 and 1324 differentially expressed genes (DEGs) were identified as canker-responsive genes in Meiwa and Newhall, respectively. Of these, 230 genes were expressed in common between both genotypes, while 564 and 1094 genes were only significantly expressed in either Meiwa or Newhall. Gene ontology (GO) annotation and Singular Enrichment Analysis (SEA) of the DEGs showed that genes related to the cell wall and polysaccharide metabolism were induced for basic defense in both Meiwa and Newhall, such as chitinase, glucanase and thaumatin-like protein. Moreover, apart from inducing basic defense, Meiwa showed specially upregulated expression of several genes involved in the response to biotic stimulus, defense response, and cation binding as comparing with Newhall. And in Newhall, abundant photosynthesis-related genes were significantly down-regulated, which may be in order to ensure the basic defense. This study revealed different molecular responses to canker disease in Meiwa and Newhall, affording insight into the response to canker and providing valuable information for the identification of potential genes for engineering canker tolerance in the future.

## Introduction

During the last decade, tremendous advancements have been achieved in the citrus industry throughout the world. However, the citrus industry worldwide suffers from an array of threats from biotic or abiotic stresses. Citrus canker caused by *Xanthomonas citri* subsp. *citri* (Xcc) is a devastating disease that has caused substantial losses in citrus-growing countries in the past decades. The canker symptoms include raised lesions on the surface of leaves, stems and fruits, with oily, water-soaked and pustule-like edges surrounded by chlorotic haloes [1], [2]. At present, the strategy for canker disease management relies on an integrated system encompassing both compatible cultural practices and phytosanitary measures, such as the eradication of inoculum sources and the application of copper-containing bactericides or antibiotics [2]. Nevertheless, since there are certain limitations associated with both cultural practices and chemical control, the issue has not been completely addressed. For example, application of copper bactericides not only increases management costs, but also raises concerns regarding environmental contamination and food safety. Furthermore, evolution of bacterial genomes over time has led to copper resistance [3]–[5]. Therefore, identification of effective compounds that can replace or supplement copper-containing chemicals is necessary. In the long run, selection or breeding of resistant cultivars may be the best solution for combating Xcc challenge in regions in which Xcc is endemic. As a fundamental step toward making these approaches possible, it is necessary to elucidate the molecular responses to Xcc invasion in the host plant.

During their long evolutionary process, plants have evolved a multitude of cellular, molecular, physiological, and biochemical alterations in order to adapt to or survive under adverse conditions, including biotic stresses caused by pathogens like Xcc. Of these alterations, molecular response at the transcriptional level has been demonstrated to be crucial for establishing a set of defense mechanisms against invading pathogens. Accumulating evidence has shown that expression of a large spectrum of genes is induced on exposure to microbial invasion in various plants [6]. The products of these genes might function to directly protect the host plant from damage caused by pathogens, or act as regulatory molecules by perceiving stress signals and transmitting them to downstream targets. These genes constitute a delicate network that plays key roles in combating pathogens. The biotic stress-induced expression of a large number of genes suggests that the nature of the biotic stress response might be more complex than expected; this is one of the reasons for the difficulty in developing a clear-cut network for the biotic stress response. As a result, although myriad molecular components responsive to pathogenic attack have been identified in a wide range of plants, the highly complex and interconnected network *per se* is still far from being fully understood. Moreover, it is worth mentioning that molecular responses may vary from plant to plant, although some parts of the responses may be common. It is therefore important to identify transcriptional changes in a given plant species under pathogenic stress in order to unravel the molecular elements that are specific to the plant itself.

Previously, researchers preferred to isolate and functionally analyze individual genes involved in the stress response. However, this is a piecemeal strategy and contributes little to a comprehensive understanding of the defense-related transcriptome that is controlled by quantitative mechanisms [7]. The advent of emerging research platforms like expression sequence tag (EST) databases, genome sequencing, and microarrays offers a good opportunity to expedite our efforts towards a better understanding of the molecular mechanisms underlying the biotic stress response. Of note, the recent availability of commercial cDNA chips provides a high-throughput approach to exploit a multitude of genes associated with many physiological processes. Transcriptomic profiling of gene expression using microarrays has been carried out in many plants under biotic stress, including *Arabidopsis* [6], [8], birch [9], sunflower [10], poplar [11], citrus [12], rice [13], [14], grape [15], and cotton [16]. Such analytical tools may reveal global gene expression changes, facilitate the elucidation of the defense response at the molecular level, and provide a significant amount of knowledge regarding potential mechanisms responsible for disease resistance, which will underpin the rationale for developing resistant germplasms via genetic engineering.

In order to isolate genes that are potentially related to canker resistance, Deng *et al.* [17] obtained 2 BAC clones containing all the features of the rice Xa21 protein, which represents a unique class of plant disease-resistance genes (*R*). Recently, Cernadas *et al.* [12] investigated the early molecular events (occurring at 4 and 48 h after inoculation) leading to canker development in sweet orange by analyzing changes in transcript levels using differential display, suppressed subtractive hybridization, and microarrays. Subsequently, Cernadas and Benedetti [18] assessed the expression patterns of cell-wall remodeling genes following Xcc infection. However, no information is yet available on a comparative transcriptome analysis between genotypes with contrasting tolerance levels to citrus canker disease. In spite of the conserved protective mechanisms among plants, resistant and susceptible genotypes may vary in their response to pathogen infection. Therefore, insightful investigation using a pair of genotypes with contrasting disease resistance phenotypes will help us better understand the molecular mechanisms underlying disease tolerance. Citrus canker has a fairly broad host range in Rutaceae and can affect many important citrus species and varieties despite the differences in the field resistance of these varieties [19]. It has been well documented that Meiwa kumquat (*Fortunella crassifolia*) is immune to Xcc, whereas Newhall navel orange (*Citrus sinensis* Osbeck) is one of the highly susceptible commercial varieties [2], [20]. In the current study, we applied the Affymetrix citrus genome GeneChip for a pairwise comparison of the gene expression profiles of Meiwa and Newhall following Xcc inoculation, in order to gain valuable insight into the mechanisms underlying canker resistance in the former.

## Results

### Comparison of canker disease development in Meiwa and Newhall

**Figure 1:**
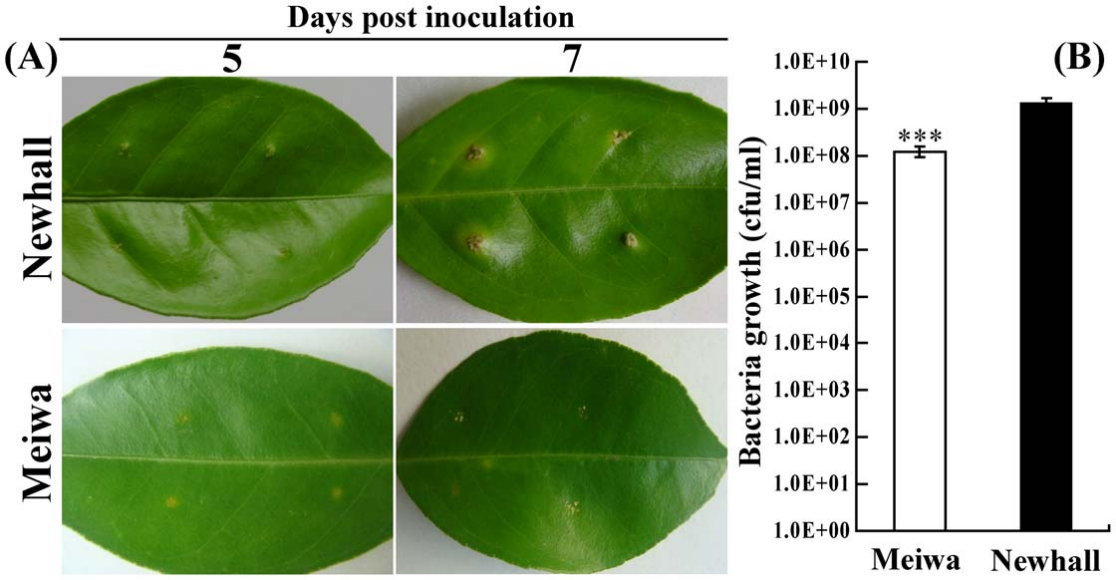
Comparison of canker disease development in ‘Meiwa’ and ‘Newhall’. (A) Leaves of ‘Meiwa’ and ‘Newhall’ were pinprick-inoculated with citrus canker bacterium and periodically observed within 7 d, and the pictures were taken at 5 and 7 days post inoculation (DPI). (B) Quantitative comparison of the bacterial population at the inoculation sites of ‘Meiwa’ and ‘Newhall’ leaves after 6 days inoculation.

To identify differences in the Xcc response between Meiwa and Newhall under our experimental conditions, the leaves of both species were pinprick-inoculated with the citrus canker bacterium and cultured in a growth chamber. As shown in Figure 1A, tiny and slightly raised lesions began to appear on the adaxial surface of Newhall leaves at 5 days post-inoculation (DPI); such lesions were not found in Meiwa. At 7 DPI, the symptoms became more conspicuous in Newhall, with typical crateriform lesions surrounded by water-soaked margins, whereas only a few tiny blister-like lesions were present in Meiwa. In addition, bacterial growth assay demonstrates that the bacterial population in the leaves of Meiwa was significantly smaller than in Newhall (Figure 1B). These results demonstrate that Meiwa was remarkably less susceptible to Xcc infection when compared with Newhall. In the current experiment, the differences in canker symptoms were apparent at 5 DPI, prior to the outbreak of the most serious symptoms. Therefore, inoculated leaves collected at 0 and 5 DPI were subjected to microarray analysis.

### Global expression profiles of Meiwa and Newhall after pinprick inoculation

**Figure 2:**
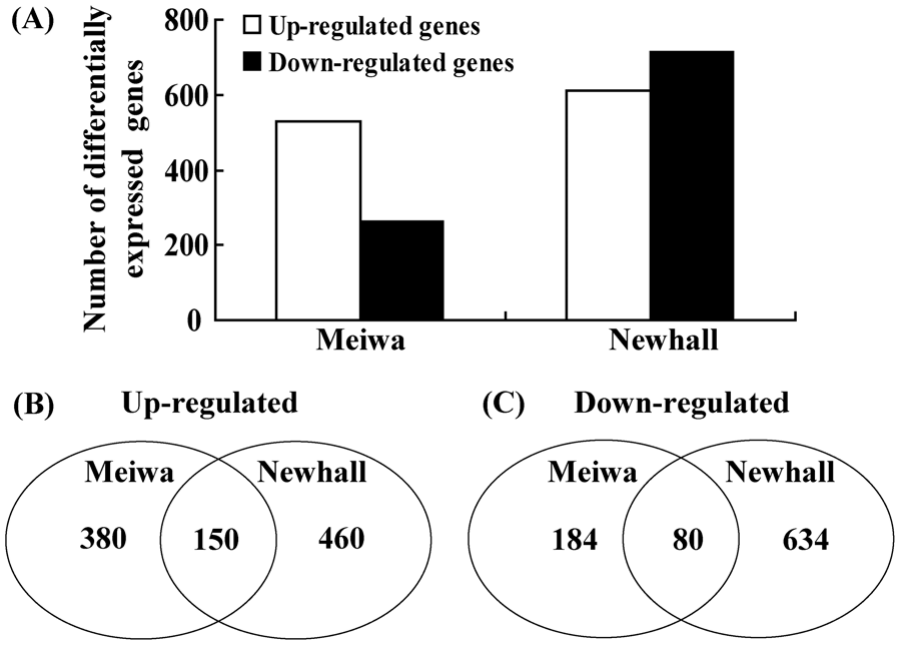
Number of differentially expressed genes in ‘Meiwa’ and ‘Newhall’ after statistical analysis. (A) Number of significantly upregulated and downregulated genes in ‘Meiwa’ and ‘Newhall’. (B–C) Venn diagram shows the number of upregulated (B) and downregulated (C) genes that are expressed in common or in special between ‘Meiwa’ and ‘Newhall’.

To reveal differences in the response to Xcc challenge between these 2 genotypes with contrasting disease tolerances and obtain new insights into the molecular mechanisms underlying canker tolerance, the transcriptomes of Meiwa and Newhall were compared by analyzing their global gene expression profiles using citrus genome Genechip. After statistical analysis, 794 and 1324 differentially expressed genes (DEGs) were identified as canker-responsive genes in Meiwa and Newhall, respectively. Among these genes, the expression of 530 genes was upregulated while that of 264 was downregulated in Meiwa. On the other hand, the expression of 610 genes was upregulated while that of 714 was downregulated in Newhall (Figure 2A). In addition, 230 out of these DEGs were identified in both Meiwa and Newhall, of which 150 showed upregulated expression while 80 showed downregulation. Moreover, of the 564 genes that showed significant expression only in Meiwa, 380 genes showed upregulated expression while 184 were downregulated following Xcc infection. In contrast, of the 1094 genes that showed significant expression only in Newhall, 460 were upregulated and 634 showed downregulation (Figure 2B–C). These data indicate that Meiwa and Newhall display noticeable differences in the Xcc response at the transcriptional level. Interestingly, the tolerant genotype, Meiwa, had significantly fewer canker-responsive genes than the canker-sensitive genotype Newhall.

All of the genes with significantly upregulated and downregulated expression levels in Meiwa and Newhall were aligned against the *Arabidopsis* database by using Citrus HarvEST software (Version 1.25), and the detailed sequence description is shown in Table S1 and Table S2. MapManBin functional annotation was also performed using the best-matched Arabidopsis Genome Initiative (AGI) number in the Plant Proteome Database (PPDB), on the basis of which the DEGs were grouped into 33 categories (Figure S1). With the exception of genes without any assignment, the main categories in both Meiwa and Newhall were related to the cell wall, secondary metabolism, hormone metabolism, stress response, miscellaneous, RNA, protein, signaling, and transport. Interestingly, expression of most of the stress-related genes was upregulated in Meiwa, but downregulated in Newhall. In addition, a total of 74 genes involved in photosynthesis showed significantly downregulated expression in Newhall. On the contrary, only 6 genes showed downregulated expression in Meiwa (Table S1 and S2).

### Verification of microarray results by semi-quantitative reverse transcription polymerase chain reaction (RT-PCR)

**Figure 3:**
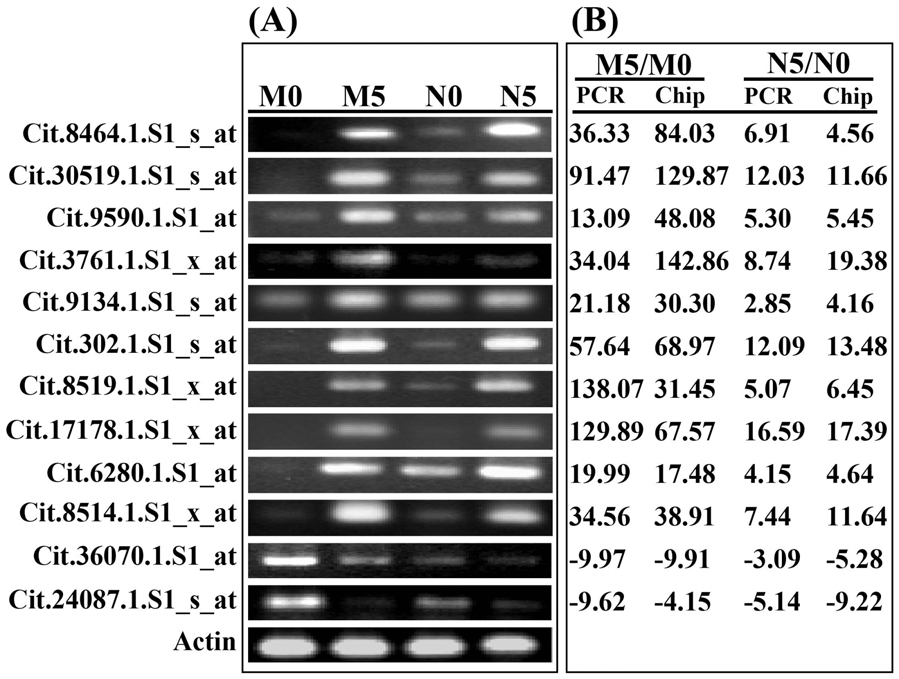
Verification of the microarray results by semi-quantitative RT-PCR. (A) RT-PCR results of the genes. The cDNA of ‘Meiwa’ and ‘Newhall’ leaves sampled at 0 (designated as M0 and N0, respectively) and 5 (designated as M5 and N5, respectively) days post inoculation (DPI) was amplified with specific primers of the selected genes, using Actin as a control. (B) Comparison of the expression ratios (M5/M0 and N5/N0) between RT-PCR analysis and the microarray data. The expression ratios were calculated by quantifying the band density using the Quantity One software.

In order to confirm the reliability of the microarray data, expression patterns of 10 up-regulated genes and 2 down-regulated genes were randomly selected and assessed by semi-quantitative RT-PCR using gene-specific primers (Table S3). These selected genes putatively encode protease inhibitor (Cit.8464.1.S1_s_at), glucanase (Cit.30519.1.S1_s_at), phenylalanine-ammonia lyase (Cit.9590.1.S1_at), pectin acetylesterase (Cit.9134.1.S1_s_at), chitinase (Cit.302.1.S1_s_at), peroxidase (Cit.8519.1.S1_x_at, Cit.8514.1.S1_x_at), protein-binding transcription regulator (Cit.6280.1.S1_at), defense-related protein (Cit.36070.1.S1_at), oxidoreductase (Cit.24087.1.S1_s_at), and unknown proteins (Cit.3761.1.S1_x_at, Cit.17178.1.S1_x_at). As shown in Figure 3, the results of RT-PCR were largely consistent with the microarray data, albeit the relative expression levels (ratio of gene levels before and after Xcc inoculation) seen with RT-PCR differed from those seen with GeneChip data in several instances (Cit.8464.1.S1_s_at, Cit.30519.1.S1_s_at, Cit.9590.1.S1_at, Cit.3761.1.S1_x_at, Cit.8519.1.S1_x_at, and Cit.17178.1.S1_x_at) in Meiwa or Newhall. Such experimental differences are not unique to this study; this phenomenon has been reported in earlier studies [21], [22] and can be attributed to intrinsic differences between the methods [22].

### Common regulated genes between Meiwa and Newhall

As mentioned above, 230 genes, 150 up-regulated and 80 down-regulated, showed significantly altered expression in both Meiwa and Newhall following Xcc infection; these are defined here as common regulated genes. These genes are logically the ones that might offer basic defense against the canker disease in citrus. It has to be pointed out that although the expression of these genes was upregulated and downregulated in common between both genotypes, we observed significantly greater changes in the expression levels of 45 upregulated and 19 downregulated genes (difference value >4.0, marked with red and blue color, respectively) in Meiwa than in Newhall (Table S4).

**Figure 4:**
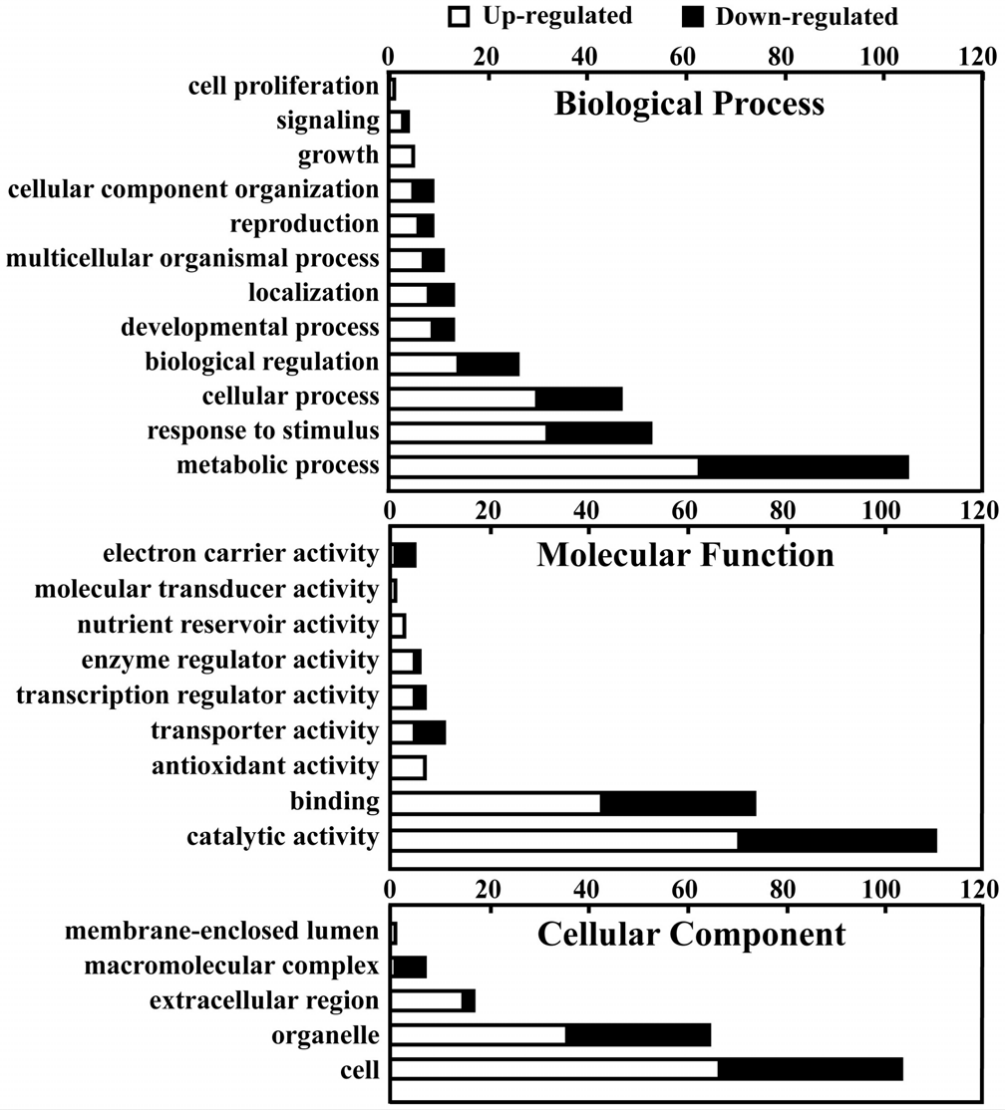
Functional categorization of the common upregulated and downregulated genes in ‘Meiwa’ and ‘Newhall’ based on the GO annotation.

To further analyze these common regulated genes, the transcripts were categorized according to their annotated function with respect to biological processes, molecular functions, and cellular components, on the basis of the blast and GO term annotation using Blast2GO software [23]. The biological processes mediated by these common regulated genes were primarily associated with metabolic processes, response to stimulus, cellular processes, biological regulation, and others (Figure 4, Table 1). Molecular functions were primarily related to catalytic and binding activity, transport, transcription regulation, enzyme regulation, electron carrier activity, antioxidant activity, nutrient reservoir activity, and molecular transduction. Interestingly, the last 3 categories were only present in the genes with upregulated expression. The cellular component categories included cell, organelle, extracellular region, macromolecular complex, and membrane-enclosed lumen. Most of these categories, such as metabolic processes, response to stimulus, antioxidant activity, transcription regulation, enzyme regulation, molecular transduction, and extracellular region, contained larger numbers of genes with typically upregulated expression than those with typically downregulated expression, whereas the electron carrier activity, transport, and macromolecular complex categories encompassed a greater proportion of genes with downregulated expression than genes with upregulated expression (Figure 4). Singular Enrichment Analysis (SEA) [24] of these GO terms was additionally carried out as described in the Materials and Methods section (Table 2). In total, 25 remarkably enriched GO terms, such as plant-type cell wall organization, polysaccharide metabolism, glucan metabolism, flavonoid metabolism, response to biotic stimulus, hydrolase activity, oxidoreductase activity, calcium ion binding, apoplast, and cell wall, were identified in the upregulated genes (Table 2, Table 3). This suggests that the genes with typically upregulated expression under these GO terms played essential roles in the response to canker disease. Interestingly, no GO terms were enriched in the genes with typically downregulated expression, which indicates that on Xcc challenge, both Meiwa and Newhall predominantly rely on the positively regulated genes to defend against invasion by the incoming pathogen.

**Table 1:** Significantly enriched GO terms (adjust *P*<0.05) of common upregulated genes in ‘Meiwa’ and ‘Newhall’ after Singular Enrichment Analysis.

Enrichment terms	Number	Adjust *P*-Value
**Biological Process**		
plant-type cell wall organization	6	9.70E-05
polysaccharide metabolic process	12	9.70E-05
polysaccharide catabolic process	5	0.005
cellular nitrogen compound metabolic process	11	0.027
cellular polysaccharide metabolic process	7	0.027
cellular glucan metabolic process	7	0.027
glucan metabolic process	7	0.027
carbohydrate metabolic process	14	0.027
response to stimulus	26	0.027
flavonoid metabolic process	5	0.027
flavonoid biosynthetic process	5	0.027
response to biotic stimulus	7	0.035
aromatic compound biosynthetic process	7	0.035
cellular amino acid derivative biosynthetic process	7	0.045
**Molecular function**		
hydrolase activity, hydrolyzing O-glycosyl compounds	11	1.50E-05
hydrolase activity, acting on glycosyl bonds	11	2.20E-05
carboxylesterase activity	7	0.00063
oxidoreductase activity	20	0.0021
oxidoreductase activity, acting on paired donors, with incorporation or reduction of molecular oxygen	6	0.0021
hydrolase activity	23	0.02
calcium ion binding	6	0.022
**Cellular component**		
extracellular region	11	1.60E-05
apoplast	5	0.0051
external encapsulating structure	6	0.021
cell wall	6	0.021

**Table 2:** Significantly enriched GO terms (adjust *P*<0.05) of specifically regulated genes in ‘Meiwa’ after Singular Enrichment Analysis.

Up-regulated genes			Down-regulated genes		
Term	Number	Adjust *P*-Value	Term	Number	Adjust *P*-Value
**Biological Process**			**Biological Process**		
lipid localization	5	7.30E-07	fatty acid metabolic process	7	0.043
carbohydrate metabolic process	39	7.30E-07	oligosaccharide metabolic process	7	0.043
polysaccharide metabolic process	22	7.30E-07	galactose metabolic process	5	0.043
chitin catabolic process	7	2.10E-05	secondary metabolic process	10	0.043
chitin metabolic process	7	2.30E-05			
glycoside metabolic process	16	2.60E-05			
cell wall macromolecule catabolic process	7	6.10E-05			
cellular carbohydrate metabolic process	24	0.00021			
aminoglycan catabolic process	7	0.00021			
response to biotic stimulus	16	0.00025			
cellular glucan metabolic process	15	0.00026			
glucan metabolic process	15	0.00026			
polysaccharide catabolic process	8	0.00026			
cell wall macromolecule metabolic process	7	0.0003			
cellular polysaccharide metabolic process	15	0.0003			
sucrose metabolic process	13	0.0003			
aminoglycan metabolic process	7	0.00041			
disaccharide metabolic process	13	0.00041			
starch metabolic process	13	0.00043			
oligosaccharide metabolic process	13	0.00046			
cell wall modification	5	0.0021			
response to other organism	13	0.0026			
multi-organism process	14	0.006			
defense response	17	0.017			
plant-type cell wall organization	5	0.022			
response to stimulus	50	0.043			
cellular amino acid derivative metabolic process	15	0.043			
cellular response to chemical stimulus	10	0.045			
**Molecular function**			**Molecular function**		
hydrolase activity, hydrolyzing O-glycosyl compounds	23	6.40E-10	transferase activity, transferring hexosyl groups	10	0.00094
hydrolase activity, acting on glycosyl bonds	23	3.00E-09	transferase activity, transferring glycosyl groups	10	0.0066
chitinase activity	8	1.50E-06	hydrolase activity, hydrolyzing O-glycosyl compounds	7	0.016
water transmembrane transporter activity	5	0.0004	phosphoprotein phosphatase activity	5	0.016
water channel activity	5	0.0004	hydrolase activity, acting on glycosyl bonds	7	0.024
carbohydrate binding	9	0.0004			
oxidoreductase activity	40	0.001			
pepsin A activity	6	0.0012			
ion binding	54	0.0067			
cation binding	54	0.0067			
**Cellular component**					
external encapsulating structure	20	1.20E-09			
cell wall	20	1.20E-09			
cytoplasmic membrane-bounded vesicle	35	0.00017			
vesicle	35	0.00017			
membrane-bounded vesicle	35	0.00017			
cytoplasmic vesicle	35	0.00017			
extracellular region	13	0.0012			
plant-type cell wall	5	0.035			

**Table 3:** Significantly enriched genes related to biotic stimulus response that are regulated in common between ‘Meiwa’ and ‘Newhall’ or specifically regulated in ‘Meiwa’.

Gene ID	Fold change in ‘Meiwa’	Fold change in ‘Newhall’	Sequence description
**Common regulated genes**			
Cit.1727.1.S1_s_at	109.8901099	4.22475708	chitinase
Cit.8464.1.S1_s_at	84.03361345	4.55996352	protease inhibitor
Cit.302.1.S1_s_at	68.96551724	13.4770889	chitinase
Cit.2116.1.S1_s_at	44.84304933	9.94035785	thaumatin-like protein
Cit.22589.1.S1_s_at	11.14827202	10.9170306	protease inhibitor
Cit.6847.1.S1_at	10.62699256	4.95540139	protein
Cit.6675.1.S1_at	4.945598417	5.44959128	protein
**Specifically regulated genes**			
Cit.11548.1.S1_at	15.50387597	#N/A	thaumatin-like protein
Cit.9703.1.S1_at	14.90312966	#N/A	β-1,3-glucanase
Cit.9706.1.S1_s_at	11.76470588	#N/A	β-1,3-glucanase
Cit.753.1.S1_x_at	10.60445387	#N/A	wound-induced protein win1
Cit.15509.1.S1_at	10.37344398	#N/A	cortical cell delineating protein expressed
Cit.1200.1.S1_s_at	9.208103131	#N/A	thaumatin-like protein
Cit.580.1.S1_x_at	7.892659826	#N/A	wound-induced protein win1
Cit.14449.1.S1_at	7.733952049	#N/A	protein
Cit.7702.1.S1_at	7.490636704	#N/A	bet v i allergen
Cit.15242.1.S1_at	7.225433526	#N/A	class iv chitinase
Cit.22667.1.S1_at	7.087172218	#N/A	UDP-glycosyltransferase
Cit.3390.1.S1_at	6.053268765	#N/A	protein
Cit.15506.1.S1_at	5.963029219	#N/A	glucosyl transferase
Cit.11721.1.S1_s_at	5.847953216	#N/A	receptor protein kinase
Cit.28117.1.S1_s_at	4.980079681	#N/A	protein
Cit.10927.1.S1_s_at	4.95049505	#N/A	harpin-induced protein

#N/A: no signals were detected.

### Differentially expressed genes specific to Meiwa

Since Meiwa is more tolerant to citrus canker disease than Newhall, DEGs present only in Meiwa may have distinctive roles in fighting against bacterial canker. A total of 564 genes, 380 with upregulated and 184 with downregulated expression, were grouped into this category, accounting for 1.87% of all the Affymetrix Citrus genome Genechip probe sets. To better understand the functions of these specifically regulated genes, BLAST analysis and GO term annotation were performed (Table S5).

**Figure 5:**
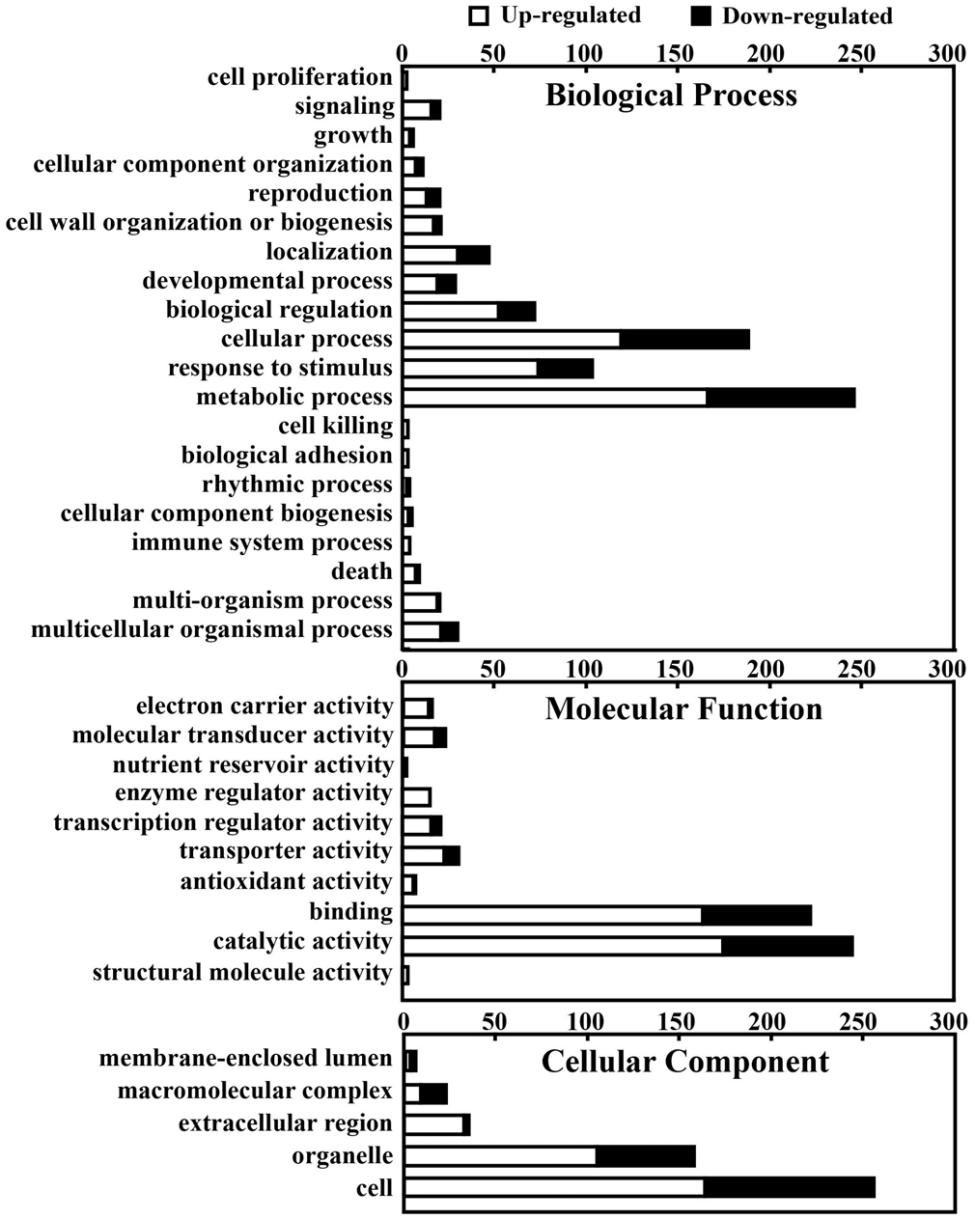
Functional categorization of 380 upregulated and 184 downregulated genes that are specifically regulated in ‘Meiwa’ based on GO annotation.

Based on the biological process, the DEGs were classified into several major groups, such as the metabolic processes, cellular processes, response to stimulus, biological regulation, and others (Table 3, Figure 5). In terms of molecular function, these genes were related to catalytic activity, binding, transport, molecular transduction, transcription regulation, enzyme regulation, and electron carrier activity, and others. The cellular component categories included cell, organelle, extracellular region, macromolecular complex, and membrane-enclosed lumen. Similar to the trend seen among the common regulated genes, the number of genes with upregulated expression in these categories was higher than that of the genes with downregulated expression. To analyze the correlation between these functional categories and the response to canker disease, SEA was again performed as described above. For genes that were specifically upregulated in Meiwa, a total of 46 significantly enriched terms were identified, including lipid localization, carbohydrate metabolism, chitin metabolism, response to biotic stimulus, glucan metabolism, defense response, hydrolase activity, chitinase activity, water channel activity, carbohydrate binding, oxidoreductase activity, cation binding, cell wall, and vesicle (Tables 4, 5 and 6). However, for the genes that are specifically downregulated in Meiwa, only 9 significantly enriched terms were identified, including fatty acid metabolism, oligosaccharide metabolism, galactose metabolism, transferase activity, hydrolase activity, phosphoprotein phosphatase activity, and others. This indicates that the enriched categories were remarkably fewer in the genes with downregulated expression than in the genes with upregulated expression. In the case of Newhall, 5 and 128 significantly enriched terms were identified specifically in the genes with upregulated and downregulated expression, respectively (Table S6). Unlike in Meiwa, the number of enriched terms in Newhall was markedly higher in the genes with downregulated expression than in the ones with upregulated expression.

**Table 4:** Significantly enriched cell wall-related genes that are regulated in common between ‘Meiwa’ and ‘Newhall’ or specifically regulated in ‘Meiwa’.

Gene ID	Fold change in ‘Meiwa’	Fold change in ‘Newhall’	Sequence description
**Common regulated genes**			
**Plant-type cell wall organization**			
Cit.30858.1.S1_at	6.406149904	5.99880024	alpha-expansin 4
Cit.14005.1.S1_s_at	7.72797527	6.978367062	Expansin
Cit.14005.1.S1_at	5.564830273	7.183908046	Expansin
Cit.8697.1.S1_at	13.42281879	5.750431282	---NA---
Cit.11232.1.S1_s_at	16.58374793	15.69858713	unnamed protein product [Vitis vinifera]
**Others**			
Cit.10363.1.S1_s_at	7.122507123	14.24501425	brassinosteroid-regulated protein bru1
Cit.302.1.S1_s_at	68.96551724	13.47708895	Chitinase
Cit.29964.1.S1_at	6.618133686	12.75510204	Protein
Cit.988.1.S1_at	7.598784195	5.920663114	Protein
Cit.30519.1.S1_s_at	129.8701299	11.65501166	xyloglucan endotransglycosylase
Cit.16722.1.S1_at	6.472491909	23.25581395	xyloglucan endotransglycosylase
**Specifically regulated genes**			
**Cell wall macromolecule metabolic**			
Cit.22311.1.S1_s_at	8.873114463	#N/A	acidic chitinase
Cit.8262.1.S1_x_at	19.92031873	#N/A	class i chitinase
Cit.8276.1.S1_x_at	34.01360544	#N/A	class i chitinase
Cit.15242.1.S1_at	7.225433526	#N/A	class iv chitinase
Cit.14913.1.S1_s_at	4.985044865	#N/A	Protein
Cit.580.1.S1_x_at	7.892659826	#N/A	wound-induced protein win1
Cit.753.1.S1_x_at	10.60445387	#N/A	wound-induced protein win1
**Cell wall modification**			
Cit.17701.1.S1_s_at	7.052186178	#N/A	pectin methylesterase
Cit.9059.1.S1_s_at	8.403361345	#N/A	pectin methylesterase
Cit.11169.1.S1_s_at	4.784688995	#N/A	pectinesterase family protein
Cit.28980.1.S1_s_at	8.802816901	#N/A	Protein
Cit.6756.1.S1_at	8.230452675	#N/A	Protein
**Plant-type cell wall organization**			
Cit.102.1.S1_s_at	12.65822785	#N/A	α-expansin 4
Cit.10687.1.S1_s_at	5.417118093	#N/A	α-expansin 4
Cit.2228.1.S1_s_at	102.0408163	#N/A	extensin-like protein
Cit.8700.1.S1_at	42.91845494	#N/A	mucin partial
Cit.8700.1.S1_s_at	83.33333333	#N/A	mucin partial
**Others**			
Cit.23827.1.S1_at	6.349206349	#N/A	at1g68400 t2e12_5
Cit.22421.1.S1_x_at	4.137360364	#N/A	miraculin-like protein 2
Cit.29356.1.S1_x_at	5.878894768	−10.9542	miraculin-like protein 2
Cit.29368.1.S1_x_at	4.638218924	−8.0095	miraculin-like protein 2
Cit.35442.1.S1_x_at	5.503577325	−26.3034	miraculin-like protein 2
Cit.72.1.S1_s_at	8.984725966	#N/A	miraculin-like protein 2
Cit.7991.1.S1_x_at	11.12347052	#N/A	miraculin-like protein 2
Cit.8083.1.S1_x_at	15.50387597	#N/A	miraculin-like protein 2
Cit.5836.1.S1_s_at	7.082152975	#N/A	---NA---
Cit.8683.1.S1_s_at	4.933399112	#N/A	non-symbiotic hemoglobin class 1
Cit.8463.1.S1_at	7.385524372	#N/A	protease inhibitor
Cit.13455.1.S1_s_at	6.349206349	#N/A	xyloglucan endotransglycosylase
Cit.2949.1.S1_s_at	4.444444444	#N/A	xyloglucan endotransglycosylase

#N/A: no signals were detected.

**Table 5:** Significantly enriched genes involved in polysaccharide metabolism that are regulated in common between ‘Meiwa’ and ‘Newhall’ or specifically regulated in ‘Meiwa’.

Gene ID	Fold change in ‘Meiwa’	Fold change in ‘Newhall’	Sequence description
**Common regulated genes**			
**Glucan metabolism**			
Cit.35196.1.S1_s_at	16.50165017	6.397952655	21 kDa protein
Cit.10363.1.S1_s_at	7.122507123	14.24501425	brassinosteroid-regulated protein BRU1
Cit.3554.1.S1_s_at	7.4019245	4.528985507	glycosyl hydrolase family 9 protein
Cit.28480.1.S1_s_at	6.353240152	7.710100231	protein
Cit.4211.1.S1_at	5.882352941	13.12335958	protein
Cit.16722.1.S1_at	6.472491909	23.25581395	xyloglucan endotransglycosylase
Cit.30519.1.S1_s_at	129.8701299	11.65501166	xyloglucan endotransglycosylase
**Others**			
Cit.20412.1.S1_s_at	67.11409396	13.96648045	basic endochitinase-like protein
Cit.1727.1.S1_s_at	109.8901099	4.224757076	chitinase
Cit.302.1.S1_s_at	68.96551724	13.47708895	chitinase
Cit.2392.1.S1_at	25.51020408	10.68376068	endo-β-1,4-glucanase
Cit.6847.1.S1_at	10.62699256	4.955401388	protein
**Specifically regulated genes**			
**Chitin metabolism**			
Cit.22311.1.S1_s_at	8.873114463	#N/A	acidic chitinase
Cit.8262.1.S1_x_at	19.92031873	#N/A	class I chitinase
Cit.8276.1.S1_x_at	34.01360544	#N/A	class I chitinase
Cit.15242.1.S1_at	7.225433526	#N/A	class IV chitinase
Cit.14913.1.S1_s_at	4.985044865	#N/A	protein
Cit.580.1.S1_x_at	7.892659826	#N/A	wound-induced protein win1
Cit.753.1.S1_x_at	10.60445387	#N/A	wound-induced protein win1
**Glucan metabolism**			
Cit.14715.1.S1_s_at	8.680555556	#N/A	acid invertase
Cit.9204.1.S1_s_at	5.941770648	#N/A	ADP-glucose pyrophosphorylase small subunit
Cit.21302.1.S1_s_at	6.172839506	#N/A	alcohol dehydrogenase
Cit.17208.1.S1_at	4.66853408	#N/A	α-amylase
Cit.9703.1.S1_at	14.90312966	#N/A	β-1,3-glucanase
Cit.9706.1.S1_s_at	11.76470588	#N/A	β-1,3-glucanase
Cit.25122.1.S1_s_at	9.652509653	#N/A	endo-β-1,4-glucanase
Cit.2945.1.S1_s_at	7.936507937	#N/A	endo-β-1,4-glucanase
Cit.30208.1.S1_at	10.41666667	#N/A	hydrolyzing o-glycosyl
Cit.9059.1.S1_s_at	8.403361345	#N/A	pectin methylesterase
Cit.11169.1.S1_s_at	4.784688995	#N/A	pectinesterase family protein
Cit.28980.1.S1_s_at	8.802816901	#N/A	protein
Cit.6756.1.S1_at	8.230452675	#N/A	protein
Cit.13455.1.S1_s_at	6.349206349	#N/A	xyloglucan endotransglycosylase
Cit.2949.1.S1_s_at	4.444444444	#N/A	xyloglucan endotransglycosylase

#N/A: no signals were detected.

**Table 6:** Significantly enriched cation binding related genes in ‘Meiwa’ specifically regulated gene cluster.

Gene ID	Fold change in ‘Meiwa’	Sequence description
**Iron ion binding**		
Cit.21723.1.S1_s_at	6.353240152	1-aminocyclopropane-1-carboxylate oxidase
Cit.30535.1.S1_s_at	5.296610169	1-aminocyclopropane-1-carboxylate oxidase
Cit.11159.1.S1_s_at	6.325110689	cytochrome P450
Cit.11965.1.S1_at	9.478672986	cytochrome P450
Cit.12598.1.S1_s_at	4.269854825	cytochrome P450
Cit.15523.1.S1_at	4.901960784	cytochrome P450
Cit.24211.1.S1_s_at	4.911591356	cytochrome P450
Cit.28253.1.S1_at	5.184033178	cytochrome P450
Cit.30299.1.S1_at	6.067961165	cytochrome P450
Cit.9904.1.S1_s_at	22.98850575	lipoxygenase
Cit.31330.1.S1_at	5.757052389	NADPH oxidase
Cit.858.1.S1_s_at	11.37656428	peroxidase 12
Cit.30798.1.S1_at	10.86956522	peroxidase precursor
Cit.5316.1.S1_at	18.86792453	protein
**Calcium ion binding**		
Cit.17208.1.S1_at	4.66853408	alpha-amylase
Cit.8157.1.S1_s_at	4.837929366	caffeoyl- 3-o-methyltransferase
Cit.24075.1.S1_at	4.980079681	CDPK-related protein kinase
Cit.31330.1.S1_at	5.757052389	NADPH oxidase
Cit.39387.1.S1_at	18.4501845	pectate lyase
Cit.858.1.S1_s_at	11.37656428	peroxidase 12
Cit.30798.1.S1_at	10.86956522	peroxidase precursor
Cit.11691.1.S1_at	10.46025105	protein
Cit.17648.1.S1_x_at	4.184100418	translationally controlled tumor protein
**Copper ion binding**		
Cit.18726.1.S1_at	6.765899865	amine oxidase
Cit.2409.1.S1_s_at	4.551661356	laccase 110a
Cit.37464.1.S1_at	4.391743522	l-ascorbate oxidase
Cit.11172.1.S1_s_at	8.223684211	pectinesterase like protein
Cit.7209.1.S1_at	15.52795031	polyphenol oxidase
**Zinc ion binding**		
Cit.21302.1.S1_s_at	6.172839506	alcohol dehydrogenase
Cit.26919.1.S1_s_at	5.341880342	ARP protein
Cit.30588.1.S1_s_at	4.387889425	ARP protein
Cit.11286.1.S1_at	5.871990605	E3 ubiquitin-protein ligase rnf149-like
Cit.20848.1.S1_at	4.076640848	protein
Cit.30654.1.S1_s_at	6.858710562	protein
Cit.38488.1.S1_at	8.244023083	protein
Cit.950.1.S1_s_at	9.900990099	protein
Cit.22054.1.S1_at	4.923682915	ring-h2 finger protein
Cit.25290.1.S1_s_at	6.105006105	ring-h2 finger protein
Cit.6340.1.S1_s_at	9.803921569	RNA binding
**Other cation binding**		
Cit.22311.1.S1_s_at	8.873114463	acidic chitinase
Cit.21938.1.S1_s_at	4.297378599	β-1,3-glucanase
Cit.9703.1.S1_at	14.90312966	β-1,3-glucanase
Cit.9706.1.S1_s_at	11.76470588	β-1,3-glucanase
Cit.24017.1.S1_at	12.01923077	cytochrome
Cit.31237.1.S1_at	5.015045135	cytochrome P450
Cit.4425.1.S1_at	14.5137881	cytochrome P450
Cit.17456.1.S1_at	6.784260516	cytochrome P450 79a2
Cit.25122.1.S1_s_at	9.652509653	endo-β-1,4-glucanase
Cit.252.1.S1_s_at	19.34235977	glycosyl hydrolase family 1 protein
Cit.30841.1.S1_s_at	12.36093943	glycosyl hydrolase family 1 protein
Cit.10770.1.S1_s_at	4.178854994	h(\+)-transporting ATPase plant fungi plasma membrane
Cit.8767.1.S1_at	6.76132522	heavy-metal-associated domain-containing expressed
Cit.6076.1.S1_s_at	6.779661017	metal ion binding
Cit.1827.1.S1_s_at	4.606172271	peroxidase
Cit.14913.1.S1_s_at	4.985044865	protein
Cit.17173.1.S1_s_at	7.496251874	urease accessory protein g

#N/A: no signals were detected.

## Discussion

### Transcriptomic characterization of Meiwa and Newhall upon Xcc infection

Comparative study is an important and effective strategy for the critical analysis of genotypes with contrasting stress tolerance. Previous studies have shown that kumquat is tolerant to citrus canker disease, while sweet orange is highly susceptible [2]. This is supported by the phenotypic observation of symptoms following pinprick inoculation of Meiwa and Newhall in our study. To better understand the molecular mechanisms underlying the difference in Xcc tolerance, and to identify the essential genes involved in canker disease tolerance, we carried out a transcriptome comparison of Meiwa and Newhall using the commercially available Citrus Affymetix GeneChip Array. The microarray data showed that the transcriptional profile of Meiwa was quite different from that of Newhall in response to canker disease. A striking difference is that a relatively smaller number of genes were induced in the tolerant Meiwa than in the susceptible Newhall following Xcc infection (Figure 2), which is consistent with the magnitude of the developing cankers in these two genotypes. Our data are consistent with the results of Taji *et al.* [25], Walia *et al.* [26], and Sun *et al.* [27] who presented data to show that on exposure to salinity, relatively fewer genes were expressed in salt-tolerant tomato, rice, or salt cress (*Thellungiella halophila*) plants than in the sensitive ones. Recently, Zheng *et al.* [28] reported that under drought stress condition, a drought-tolerant maize inbred line had fewer drought-responsive genes than did a drought-sensitive line. One of the explanations for this phenomenon might be that tolerant genotypes exhibit a limited molecular response at the transcriptional level because they suffer from a relatively lower degree of stress injury than the susceptible ones. Notably, in Meiwa, the number of genes with upregulated expression was approximately twice that of the genes with downregulated expression. In contrast, in Newhall, genes with upregulated expression were fewer than those with downregulated expression. This result indicates that Meiwa might mainly deploy positive regulation in response to canker disease, and that negative regulation is predominant in Newhall.

MapManBin classification [29] indicates that the expression of a large number of photosynthesis-related genes was downregulated in Newhall, including those encoding proteins involved in photosystem I (PSI), photosystem II (PSII), ATP synthase, and the Calvin cycle. In contrast, the expression of only a few genes involved in photosynthesis was downregulated in Meiwa. Downregulation of the expression of photosynthesis-related genes showed good agreement with the findings in previous studies, indicating that the expression of many photosynthetic genes was repressed on encountering a biotic attack [30]–[32]. These results suggest that photosynthetic function in the host plant is compromised when biotic attack results in morphological changes, such as the establishment of canker in the leaves. In this context, the larger number of genes repressed in the susceptible genotype (Newhall) is consistent with the more serious canker symptoms observed, in comparison with the severity of the symptoms in the tolerant genotype. One explanation for this is that the susceptible genotype may require a larger investment in defense needs by the reallocation of nitrogen from the photosynthetic pathway to the defense machinery [32]–[34]. However, rather than being the cause of the tolerance or susceptibility mechanism, the repressed expression of photosynthesis-related genes under such circumstances may merely reflect the influence of the biotic challenge on the host plant.

Despite the aforementioned differences, Meiwa and Newhall still share a large number of common regulated genes following Xcc infection. These genes may be necessary for the response to biotic stress and may play basic roles in defense against Xcc. This is logical, as plants have evolutionarily developed conserved defense machinery against invading pathogens irrespective of their stress tolerance capacity, suggesting that they may exhibit the same subsets of gene expression and signaling pathways under adverse environmental stresses. This implies that the expression of these genes can be also induced in other citrus species upon bacterial invasion. Although we did not try to identify the expression patterns of common regulated genes in other cultivars, it is interesting to note that several common regulated genes, such as cytochrome P450, xyloglucan endotransglycosylase, phenylalanine-ammonia lyase, expansin, peroxidase, and chitinase-related genes, have been shown to be induced in other citrus cultivars, such as ‘Pêra’ and ‘Cristal’, following Xcc inoculation [12]. The identification of common regulated genes is not unique to this study; it has been reported in other stressful conditions. For instance, Zheng *et al.* [28] presented data to show that drought-tolerant and drought-sensitive maize lines expressed a common set of genes in response to drought stress.

DEGs that are specifically present in Meiwa are more important than the common regulated genes, as the former might provide valuable information on the molecular basis of canker tolerance in this genotype. We used GO term enrichment analysis to gain more insight into these genes, as it is traditionally an efficient strategy to analyze the representation of genes under different categories by comparing gene expression profiles to the background [24]. GO term enrichment analysis revealed a remarkable difference in gene distribution frequency between Meiwa and Newhall: the former has a larger number of genes enriched in the upregulated gene cluster, while the latter had more categories of genes enriched in the downregulated cluster.

### Significantly enriched genes in response to biotic stimulus

According to the GO term enrichment analysis, 7 and 16 genes responsive to biotic stimulus were significantly enriched in the common regulated and Meiwa-specifically regulated genes, respectively (Table 1 and Table 2). Based on functional annotation, these genes, encoding proteins such as chitinase, proteinase inhibitor, thaumatin-like protein, β-1, 3-glucanase, wound-induced protein win1, receptor protein kinase, and other unknown proteins, are directly involved in responses to biotic stimuli. Proteinase inhibitors (PIs) belong to the PR6 family, which is widely distributed in the plant kingdom [35], [36]. Several reports have shown that plant PIs are an essential part of the natural defense against pathogens [37]–[39]. These results provide a clue that PIs may potentially perform specific functions in response to citrus canker. Thaumatin-like proteins (TLPs), categorized under the PR5 family, have been shown to accumulate when plants are attacked by pathogens [35]. In addition, *in vitro* bioassays have shown that TLPs possess antifungal activity [40]. Datta *et al.* [41] presented data to show that the overexpression of a TLP gene conferred resistance to sheath blight disease in transgenic rice. These data led us to hypothesize that the induction of TLP genes might be an integral part of the defense machinery against canker in Meiwa. Receptor-like kinases act on the recognition of pathogen-associated molecular patterns, such as bacterial flagellin, which trigger immunity and effector-mediated immune responses [42], [43]. In the present study, a receptor kinase was abundantly enriched in the subset of genes specifically regulated in Meiwa, indicating that Meiwa might activate downstream defenses in a more efficient manner. Although the functions of these genes have not been verified herein, we can speculate that these genes are closely involved in the canker resistance of Meiwa, in light of previous studies.

### The expression levels of cell wall and polysaccharide metabolism-related genes change markedly in Meiwa upon canker infection

Among both common regulated and Meiwa-specific genes, an array of genes related to cell wall and polysaccharide metabolism was significantly enriched. The plant cell wall plays an important role in basal defense, as it is the primary region of the host-pathogen interaction and constitutes the first physical barrier to limit pathogen colonization [44]–[47]. Enriched cell wall-related genes, such as expansin and xyloglucan endotransglycosylase (XET), are responsible for plant cell wall organization, macromolecule metabolism, and cell wall modification (Table 4). Expansins are a family of proteins found within plant cell walls that are responsible for cell wall disassembly, cell separation, and cell expansion [48]. Genes coding for expansins have been shown to be responsive to biotic or abiotic stresses [49]–[52]. Recently, expansin genes were found to be induced when citrus plants were challenged with Xcc or Huanglongbing pathogen [12], [53]. Up-regulation of the expansin gene suggests that following Xcc inoculation, host plants may accumulate a larger amount of this protein, which functions as a cell wall loosening agent to increase cell wall extensibility [51]. XET catalyzes the cleavage of the xyloglucan backbone, a major structural hemicellulose polysaccharide in the primary cell wall, to form secondary cell walls. The induction of these genes indicates that modification of cell wall flexibility may be a crucial protective strategy in Meiwa to limit pathogen invasion or spread in the internal tissues.

It has been well documented that high-molecular-weight polysaccharides such as chitin and peptidoglycan are the major components of the cell wall of pathogens. Here, Xcc infection led to a significant induction of genes involved in chitin and glucan metabolism in Meiwa, including chitinase, endo-β-1, 4-glucanase (EGase), and β-1, 3-glucanase (Table 5). This is consistent with the work of Cernadas *et al.* [12], who found that inoculation with the citrus canker pathogen enhanced the transcriptional levels of these genes in sweet orange. Chitinase (EC 3.2.1.14) hydrolyzes the β-1, 4-glycoside bond present in the chitin polymer to release chitin fragments, such as chitooligosaccharides or chitin oligomers, from cell walls and thereby activate plant innate immunity [54]–[56]. The presence of abundant chitinase genes in Meiwa suggests that the degradation of the cell wall of invading pathogens is more extensive in this genotype, leading to inhibition of pathogen proliferation and the spread or induction of systemic defense. Moreover, EGase functions in cell wall loosening, which is important for expansion or major cell wall disruption [57], [58]. β-1, 3-glucanase, hydrolyzing the 1, 3-β-d-glucosidic linkages of β-1, 3-glucan, has been shown to play a crucial role in plant pathogen defense [59]–[61].

### Genes related to cation binding are prominently enriched in Meiwa upon Xcc infection

Among the genes specifically regulated in Meiwa, as many as 54 genes were significantly enriched in the cation-binding category, including those involved in the binding of iron, calcium, copper, zinc, and other unknown ions (Table 2 and Table 6). In contrast, this category of genes was not enriched among the genes specifically regulated in Newhall. This finding implies that the cation-binding process may be, at least in part, responsible for the difference in canker tolerance between these two genotypes. Copper ion binding is potentially of interest, because copper-containing bactericides have been extensively applied to control citrus canker disease [2], [62]–[64]. Very recently, Yuan *et al.* [65] reported that the copper level is a key determinant of the defense response to *Xanthomonas oryzae* causing blight in rice. The induction of copper ion binding suggests that copper redistribution may be modified in Meiwa in order to protect the host plant from Xcc bacterial invasion, as has been documented by Yuan *et al.* [65]. Apart from copper ion binding, there are 14 genes related to iron ion binding, 7 of which encode cytochrome P450 (Table 6). Iron is an important micronutrient for virtually all living organisms, and iron homeostasis commonly occurs in pathogen-host interactions. Bacteria need to acquire iron from the host for their own survival, and the host can limit bacterial pathogen invasion through an iron-withholding model [66]. Enrichment of these genes in Meiwa demonstrates that the uptake of iron ions by bacteria from the host plant may be efficiently restrained, leading to growth arrest of the invading pathogens. Cytochrome P450s, a group of heme-containing enzymes that are ubiquitously present in bacteria and plants [67], have been shown to regulate the biosynthesis of defense-related compounds [68], [69]. Calcium ion binding probably plays an important signaling role in response to canker disease, because calcium is a well-known second messenger in numerous plant signaling pathways [70]. However, the physiological or molecular relevance of zinc ion binding and that of other cations remains to be determined, as limited information is available so far on their activation upon pathogen attack.

Taken together, a comparative transcriptomic analysis of Meiwa and Newhall in this study reveals that they differ greatly in the molecular response to citrus canker, which may partially explain their phenotypical variation with regard to disease tolerance. When challenged with Xcc bacteria, expression of genes involved in polysaccharide metabolism, biotic stimulus response, cell wall strengthening, and cation binding was altered, thereby promoting the production/synthesis of a large spectrum of second metabolites and modifying ion homeostasis. These biological processes may work cooperatively to limit bacterial penetration, proliferation, spread, and growth, conferring canker tolerance. By contrast, in Newhall only a few basal responsive proteins such as chitinase, glucanase, and thaumatin-like protein were activated, leading to the production of a limited amount of relevant products that function to protect the host against the canker pathogen. In addition, Xcc attack repressed the expression of several genes associated with photosynthesis in Newhall. The data presented herein revealed the molecular mechanisms underlying the contrasting canker tolerance between Meiwa and Newhall, and the Meiwa-specific regulated genes hold great potential for engineering canker tolerance in the future. The next challenge is to narrow down the genes screened in this study based on expression patterns and to finally exploit and functionally identify the genes that are truly responsible for the canker tolerance. In addition, creation of transgenic plants with enhanced canker tolerance using the genes tapped from this study will be of paramount significance for providing novel germplasms that can be integrated into citrus breeding pipeline in the long run.

## Materials and Methods

### Plant materials and bacterial strains

Leaves were collected from uniform and healthy summer flushes of 15-year-old Meiwa (*Fortunella crassifolia*) and Newhall (*Citrus sinensis* Osbeck) plants grown in the same orchard in the Citrus Research Institute, Huazhong Agricultural University (Wuhan, China). The primary source of the inoculum used in this study was Xcc strain A (X02-007), provided by Prof. Hong Ni (Huazhong Agricultural University). The bacteria were maintained at 28°C in SPA medium containing sucrose 20 g/l, peptone 5 g/l, K_2_HPO_4_ 0.5 g/l, MgSO_4_•7 H_2_O 0.25 g/l, and agar 15 g/l (pH 7.2–7.4).

### Pinprick inoculation of leaves and sampling

The bacterial strain was cultured in liquid SPA medium at 28°C and shaken overnight at 200 rpm, then collected by centrifugation and re-suspended in the medium at a concentration of about 10^8^ cells/ml before inoculation. The collected leaves were washed with distilled water and then subjected to inoculation on the abaxial side using an inoculating needle (0.5 mm in diameter). Four inoculations, each composed of 5 pricks, were made on both sides of the midvein, and a 10-µl aliquot of the bacterial suspension was dropped onto each prick. Following inoculation, the leaves were placed on wet filter paper in Petri dishes, which were then sealed with parafilm to maintain high humidity for bacterial growth. The Petri dishes were kept at 28°C in a plant growth chamber for the indicated periods. Initiation of symptoms was scored within a 7-d cycle. The leaves were immediately immersed in liquid nitrogen and stored at −80°C till use. Leaves sampled at 0 and 5 DPI were used for microarray analysis.

### Bacterial growth assay

Bacterial population in the inoculated sites collected at 6 DPI (after canker appearance) was examined based on earlier report [71]. In brief, the inoculated sites of same size were disinfected with 2% (v/v) sodium hypochlorite for 10 s and 75% ethanol for 3 min. The leaf discs were then ground in sterile distilled water, followed by dilution and spread on SPA medium. After an incubation for 2 d at 28°C the number of colonies was counted in order to calculate the colony-forming units (cfu), expressed as cfu/ml.

### Total RNA isolation, probe preparation, and microarray hybridization

Total RNA was isolated from samples collected at 0 and 5 DPI using the Trizol reagent (Invitrogen, Carlsbad, CA) according to the supplier's recommendations. The RNA samples were treated with amplification-grade DNase I (Takara, Dalian, China) at 37°C to remove any contaminant genomic DNA. Gene expression profiles of Meiwa and Newhall before (0 DPI) and after (5 DPI) Xcc inoculation was analyzed by the Affymetrix Citrus Genome GeneChip one-cycle target labeling and control kit (Affymetrix, Santa Clara, CA) according to the manufacturer's instructions; this was done by Gene Technology Company Limited (Shanghai, China). For GeneChip analysis, 10 µg of total RNA was first reverse transcribed into double-stranded cDNA using a T7-Oligo(dT) promoter primer, then transcribed to complementary RNA (cRNA) *in vitro* in the presence of T7 RNA polymerase and a biotinylated nucleotide analog/ribonucleotide mix for cRNA amplification and biotin labeling. The resultant biotinylated cRNA targets, which were labeled with either Cy5 (5 DPI samples) or Cy3 (0 DPI samples), were then cleaned up, fragmented, and hybridized with the Citrus Genome GeneChip Array, which contained 30,171 probe sets representing up to 33,879 citrus transcripts based on EST sequences obtained from several citrus species and citrus hybrids. According to the published sweet orange genome (version 1, http://www.phytozome.net), the transcripts on the array account for 73.4% of the whole genome. Cy5-labeled cRNAs were hybridized with Cy3-labeled cRNAs for each genotype. Hybridization was performed on each of the materials tested with 2 biological replicates and two technical replicates (dye-swap).

### Data analysis

After the washing procedure was completed, the probe array was scanned using the Affymetrix GeneChip Scanner 3000. The images were analyzed using the Affymetrix GeneChip Operating Software (GCOS 1.4) to generate raw data, which was saved as CEL files. The CEL files were then imported into Bioconductor system (R software) using the Affy package for quantile normalization to obtain Robust Multi-array Average (RMA) data containing the expression values. For statistical analysis of differentially expressed genes between Meiwa and Newhall, the *RankPord* package in R software [72] was used to calculate the number of false-positive predictions (FPP), which is also known as the false discovery rate (FDR) [73]. Probe sets with an FDR≤0.5 and a 4-fold change were considered as differentially expressed genes at a statistically significant level. DEGs in Meiwa and Newhall were functionally annotated using the Citrus HarvEST software (Version 1.25, http://harvest.ucr.edu/, University of California) by aligning the consensus sequences of all probe sets to the sequences in the Arabidopsis database, and the MapManBin [29] functional categorization was carried out online in the Plant Proteome Database (PPDB) [74] using the best matched AGI number. For further analysis of the common regulated genes and Meiwa-specifically regulated genes, Blast analysis and GO term annotation were carried out using Blast2GO software [23]. GO terms for each of the 3 main categories, biological process, molecular function, and cellular component, were obtained from sequence similarity using default parameters. To analyze GO term enrichment of significant DEGs, SEA was performed online through agriGO (http://bioinfo.cau.edu.cn/agriGO), a GO analysis tool kit for the agricultural community [24]. In brief, the probe ID numbers of common regulated genes or specifically regulated genes were first uploaded into the agriGO, and the Citrus Affymetrix Genome Array was selected as the background. Thereafter, statistical *P*-values were calculated using the hypergeometric method, and multiple comparison correction was done using the Benjamini-Yekutieli method to adjust *P*-values [75]. GO terms with an adjusted *P* value<0.05 were considered to be significantly enriched in the leaves of Meiwa and Newhall before and after inoculation.

### Semi-quantitative RT-PCR analysis

Semi-quantitative RT-PCR was employed to verify the microarray results. The same RNA samples tested in the hybridization experiments were used for cDNA synthesis using the ReverTra Ace-α-™ kit (Toyobo, Osaka, Japan) following the manufacturer's instructions. Primers specific to 10 upregulated and 2 downregulated genes were designed using the Primer Premier 5 software (PRIMER Biosoft International, Palo Alto, CA) based on the consensus sequences (Table S3). The amplification was carried out in a thermal cycler (Bio-Rad, Hercules, CA) with a program of 28 cycles of 30 s at 94°C, 30 s at 55°C and 45 s at 72°C. The same cDNA was amplified with primers specific to an actin gene, which was used as an internal positive control. Band density was quantified using Quantity One Software (Version 4.6.2, Bio-Rad). PCR amplification of each gene was performed in triplicate.

## Supporting Information

Figure S1 **MapManbin classification of differentially expressed genes in ‘Meiwa’ and ‘Newhall’.**
(TIF)

Table S1 **List and Mapman analysis of differentially expressed genes in ‘Meiwa’.**
(XLS)

Table S2 **List and Mapman analysis of differentially expressed genes in ‘Newhall’.**
(XLS)

Table S3 **Sequences of the specific primers used for the semi-quantitative RT-PCR analysis.**
(XLS)

Table S4 **The common upregulated (150) or downregulated genes (80) in ‘Meiwa’ and ‘Newhall’ after Xcc infection, among which 45 upregulated and 19 downregulated in ‘Meiwa’ showed significantly higher fold change than ‘Newhall’ (difference value >4, marked with color).**
(XLS)

Table S5 **The specifically upregulated (380) or downregulated (184) genes in ‘Meiwa’ after Xcc infection.**
(XLS)

Table S6 **Significantly enriched GO terms of the specifically regulated genes in ‘Newhall’ after Singular Enrichment analysis.**
(XLS)

## References

[1] Schubert TS, Rizvi SA, Sun X, Gottwald TR, Graham JH (2001). Meeting the challenge of eradicating citrus canker in Florida-again.. Plant Diseas.

[2] Das AK (2003). Citrus canker - A review.. Journal of Applied Horticulture.

[3] Stall RE, Loschke DC, Jones JB (1986). Linkage of copper resistance and avirulence loci on a self-transmissible plasmid in *Xanthomonas campestris* pv. *vesicatoria*.. Phytopathology.

[4] Behlau F, Canteros BI, Minsavage GV, Jones JB, Graham JH (2011). Molecular Characterization of Copper Resistance Genes from *Xanthomonas citri* subsp *citri* and *Xanthomonas alfalfae* subsp *citrumelonis*.. Applied and Environmental Microbiology.

[5] Behlau F, Jones JB, Myers ME, Graham JH (2012). Monitoring for resistant populations of *Xanthomonas citri* subsp *citri* and epiphytic bacteria on citrus trees treated with copper or streptomycin using a new semi-selective medium.. European Journal of Plant Pathology.

[6] Schenk PM, Kazan K, Wilson I, Anderson JP, Richmond T (2000). Coordinated plant defense responses in Arabidopsis revealed by microarray analysis.. Proceedings of the National Academy of Sciences of the United States of America.

[7] Eulgem T (2005). Regulation of the Arabidopsis defense transcriptome.. Trends in Plant Science.

[8] Tao Y, Xie Z, Chen W, Glazebrook J, Chang HS (2003). Quantitative nature of Arabidopsis responses during compatible and incompatible interactions with the bacterial pathogen *Pseudomonas syringae*.. Plant Cell.

[9] Wright DP, Johansson T, Le Quéré A, Söderström B, Tunlid A (2005). Spatial patterns of gene expression in the extrametrical mycelium and mycorrhizal root tips formed by the ectomycorrhizal fungus *Paxillus involutus* in association with birch (*Betula pendula*) seedlings in soil microcosms.. New Phytologist.

[10] Alignan M, Hewezi T, Petitprez M, Dechamp-Guillaume G, Gentzbittel L (2006). A cDNA microarray approach to decipher sunflower (*Helianthus annuus*) responses to the necrotrophic fungus *Phoma macdonaldii*.. New Phytologist.

[11] Rinaldi C, Kohler A, Frey P, Duchaussoy F, Ningre N (2007). Transcript profiling of poplar leaves upon infection with compatible and incompatible strains of the foliar rust *Melampsora larici-populina*.. Plant Physiology.

[12] Cernadas RA, Camillo LR, Benedetti CE (2008). Transcriptional analysis of the sweet orange interaction with the citrus canker pathogens *Xanthomonas axonopodis* pv. *citri* and *Xanthomonas axonopodis* pv. *aurantifolii*.. Molecular Plant Pathology.

[13] Fujiwara S, Tanaka N, Kaneda T, Takayama S, Isogai A (2004). Rice cDNA microarray-based gene expression profiling of the response to flagellin perception in cultured rice cells.. Molecular plant-Microbe Interactions.

[14] Qiu D, Xiao J, Xie W, Liu H, Li X (2008). Rice gene network inferred from expression profiling of plants overexpressing OsWRKY13, a positive regulator of disease resistance.. Molecular Plant.

[15] Albertazzi G, Milc J, Caffagni A, Francia E, Roncaglia E (2009). Gene expression in grapevine cultivars in response to Bois Noir phytoplasma infection.. Plant Science.

[16] Miao WG, Wang XB, Song CF, Wang Y, Ren YH (2010). Transcriptome analysis of *Hpa1_Xoo_* transformed cotton revealed constitutive expression of genes in multiple signalling pathways related to disease resistance.. Journal of Experimental Botany.

[17] Deng Z, Gmitter FG (2003). Cloning and characterization of receptor kinase class disease resistance gene candidates in citrus.. Theoretical and Applied Genetics.

[18] Cernadas RA, Benedetti CE (2009). Role of auxin and gibberellin in citrus canker development and in the transcriptional control of cell-wall remodeling genes modulated by *Xanthomonas axonopodis* pv. *citri*.. Plant Science.

[19] Gottwald TR, Graham JH, Timmer LW, Garnsey SM, Graham H (2000). Canker.. *Compendium of citrus diseases*, 2nd edtion.

[20] Wang Y, Fu XZ, Liu JH, Hong N (2011). Differential structure and physiological response to canker challenge between ‘Meiwa’ kumquat and ‘Newhall’ navel orange with contrasting resistance.. Scientia Horticulturae.

[21] López C, Soto M, Restrepo S, Piégu B, Cooke R (2005). Gene expression profile in response to *Xanthomonas axonopodis* pv. *manihotis* infection in cassava using a cDNA microarray.. Plant Molecular Biology.

[22] Gandía M, Conesa A, Ancillo G, Gadea J, Forment J (2007). Transcriptional response of *Citrus aurantifolia* to infection by Citrus tristeza virus.. Virology.

[23] Conesa A, Gotz S, Garcia-Gomez JM, Terol J, Talon M (2005). Blast2GO: a universal tool for annotation, visualization and analysis in functional genomics research.. Bioinformatics.

[24] Du Z, Zhou X, Ling Y, Zhang Z, Su Z (2010). AgriGO: a GO analysis toolkit for the agricultural community.. Nucleic Acids Research.

[25] Taji T, Seki M, Satou M, Sakurai T, Kobayashi M (2004). Comparative genomics in salt tolerance between Arabidopsis and Arabidopsis-related halophyte salt cress using Arabidopsis microarray.. Plant Physiology.

[26] Walia H, Wilson C, Condamine P, Liu X, Ismail AM (2005). Comparative transcriptional profiling of two contrasting rice genotypes under salinity stress during the vegetative growth stage.. Plant Physiology.

[27] Sun W, Xu XN, Zhu HS, Liu AH, Liu L (2010). Comparative transcriptomic profiling of a salt-tolerant wild tomato species and a salt-sensitive tomato cultivar.. Plant and Cell Physiology.

[28] Zheng J, Fu JJ, Gou MY, Huai JL, Liu YJ (2010). Genome-wide transcriptome analysis of two maize inbred lines under drought stress.. Plant Molecular Biology.

[29] Thimm O, Blasing O, Gibon Y, Nagel A, Meyer S (2004). MAPMAN: a user-driven tool to display genomics data sets onto diagrams of metabolic pathways and other biological processes.. Plant Journal.

[30] Zou J, Rodriguez-Zas S, Aldea M, Li M, Zhu J (2005). Expression profiling soybean response to *Pseudomonas syringae* reveals new defense-related genes and rapid HR-specific downregulation of photosynthesis.. Molecular Plant-Microbe Interactions.

[31] Berger S, Benediktyova Z, Matous K, Bonfig K, Mueller MJ (2007). Visualization of dynamics of plant-pathogen interaction by novel combination of chlorophyll fluorescence imaging and statistical analysis: differential effects of virulent and avirulent strains of *P. syringae* and of oxylipins on *A. thaliana*.. Journal of Experimental Botany.

[32] Bilgin DD, Zavala JA, Zhu J, Clough SJ, Ort DR (2010). Biotic stress globally downregulates photosynthesis genes.. Plant Cell and Environment.

[33] Evans JR (1989). Photosynthesis and nitrogen relationships in leaves of C_3_ plants.. Oecologia.

[34] Paul MJ, Foyer CH (2001). Sink regulation of photosynthesis.. Journal of Experimental Botany.

[35] Jwa NS, Agrawal GK, Tamogami S, Yonekura M, Han O (2006). Role of defense/stress-related marker genes, proteins and secondary metabolites in defining rice self-defense mechanisms.. Plant Physiology and Biochemistry.

[36] Huang YM, Xiao BZ, Xiong LZ (2007). Characterization of a stress responsive proteinase inhibitor gene with positive effect in improving drought resistance in rice.. Planta.

[37] Botella MA, Xu Y, Prabha TN, Zhao Y, Narasimhan ML (1996). Differential expression of soybean cysteine proteinase inhibitor genes during development and in response to wounding and methyl jasmonate.. Plant Physiology.

[38] Solomon M, Belenghi B, Delledonne M, Menachem E, Levine A (1999). The involvement of cysteine proteases and protease inhibitor genes in the regulation of programmed cell death in plants.. Plant Cell.

[39] Rakwal R, Kumar Agrawal G, Jwa NS (2001). Characterization of a rice (*Oryza sativa* L.) Bowman-Birk proteinase inhibitor: tightly light regulated induction in response to cut, jasmonic acid, ethylene and protein phosphatase 2A inhibitors.. Gene.

[40] Hejgaard J, Jacobsen S, Svendsen I (1991). Two antifungal thaumatin-like proteins from barley grains.. FEBS Letters.

[41] Datta K, Velazhahan R, Oliva N, Ona I, Mew T (1999). Over-expression of the cloned rice thaumatin-like protein (PR-5) gene in transgenic rice plants enhances environmental friendly resistance to *Rhizoctonia solani* causing sheath blight disease.. Theoretical and Applied Genetics.

[42] Chisholm ST, Coaker G, Day B, Staskawicz BJ (2006). Host-microbe interactions: shaping the evolution of the plant immune response.. Cell.

[43] Gómez-Gómez L, Boller T (2000). FLS2: an LRR receptor–like kinase involved in the perception of the bacterial elicitor flagellin in *Arabidopsis*.. Molecular Cell.

[44] Vorwerk S, Somerville S, Somerville C (2004). The role of plant cell wall polysaccharide composition in disease resistance.. Trends in Plant Science.

[45] Cantu D, Vicente AR, Labavitch JM, Bennett AB, Powell ALT (2008). Strangers in the matrix: plant cell walls and pathogen susceptibility.. Trends in Plant Science.

[46] Hématy K, Cherk C, Somerville S (2009). Host-pathogen warfare at the plant cell wall.. Current Opinion in Plant Biology.

[47] Lagaert S, Belien T, Volckaert G (2009). Plant cell walls: Protecting the barrier from degradation by microbial enzymes.. Seminars in Cell & Developmental Biology.

[48] Cosgrove DJ (2000). Loosening of plant cell walls by expansins.. Nature.

[49] Hiwasa K, Rose JKC, Nakano R, Inaba A, Kubo Y (2003). Differential expression of seven alpha-expansin genes during growth and ripening of pear fruit.. Physiologia Plantarum.

[50] Balestrini R, Cosgrove DJ, Bonfante P (2005). Differential location of α-expansin proteins during the accommodation of root cells to an arbuscular mycorrhizal fungus.. Planta.

[51] Xu J, Tian J, Belanger FC, Huang BR (2007). Identification and characterization of an expansin gene *AsEXP1* associated with heat tolerance in C_3_
*Agrostis* grass species.. Journal of Experimental Botany.

[52] Ding X, Cao Y, Huang L, Zhao J, Xu C (2008). Activation of the indole-3-acetic acid-amido synthetase GH3–8 suppresses expansin expression and promotes salicylate- and jasmonate-independent basal immunity in Rice.. Plant Cell.

[53] Albrecht U, Bowman KD (2008). Gene expression in *Citrus sinensis* (L.) Osbeck following infection with the bacterial pathogen *Candidatus* Liberibacter asiaticus causing Huanglongbing in Florida.. Plant Science.

[54] Punja ZK, Zhang YY (1993). Plant chitinases and their roles in resistance to fungal diseases.. Journal of Nematology.

[55] Kasprzewska A (2003). Plant chitinases—Regulation and function.. Cellular & Molecular Biology Letters.

[56] Wan J, Zhang XC, Stacey G (2008). Chitin signaling and plant disease resistance.. Plant Signaling & Behavior.

[57] Loopstra CA, Mouradov A, Vivian-Smith A, Glassick TV, Gale BV (1998). Two pine endo-β-1, 4-glucanases are associated with rapidly growing reproductive structures.. Plant Physiology.

[58] Bourquin V, Nishikubo N, Abe H, Brumer H, Denman S (2002). Xyloglucan endotransglycosylases have a function during the formation of secondary cell walls of vascular tissues.. Plant Cell.

[59] Jongedijk E, Tigelaar H, Van Roekel JSC, Bres-Vloemans SA, Dekker I (1995). Synergistic activity of chitinases and β-1, 3-glucanases enhances fungal resistance in transgenic tomato plants.. Euphytica.

[60] Lusso M, Kuc J (1996). The effect of sense and antisense expression of the PR-N gene for β-1, 3-glucanase on disease resistance of tobacco to fungi and viruses.. Physiological and Molecular Plant Pathology.

[61] Enrique R, Siciliano F, Favaro MA, Gerhardt N, Roeschlin R (2010). Novel demonstration of RNAi in citrus reveals importance of citrus callose synthase in defence against *Xanthomonas citri* subsp. *citri*.. Plant Biotechnology Journal.

[62] Graham JH, Gottwald TR, Cubero J, Achor DS (2004). *Xanthomonas axonopodis* pv. *citri*: factors affecting successful eradication of citrus canker.. Molecular Plant Pathology.

[63] Behlau F, Belasque J, Bergamin A, Graham JH, Leite RP (2008). Copper sprays and windbreaks for control of citrus canker on young orange trees in southern Brazil.. Crop Protection.

[64] Behlau F, Belasque J, Graham JH, Leite RP (2010). Effect of frequency of copper applications on control of citrus canker and the yield of young bearing sweet orange trees.. Crop Protection.

[65] Yuan M, Chu ZH, Li XH, Xu CG, Wang SP (2010). The bacterial pathogen *Xanthomonas oryzae* overcomes rice defenses by regulating host copper redistribution.. Plant Cell.

[66] Johnson L (2008). Iron and siderophores in fungal-host interactions.. Mycological Research.

[67] Hwang IS, Hwang BK (2010). Role of the pepper cytochrome P450 gene *CaCYP450A* in defense responses against microbial pathogens.. Planta.

[68] Schuler MA, Werck-Reichhart D (2003). Functional genomics of P450s.. Annual Review of Plant Biology.

[69] Kim YC, Kim SY, Paek KH, Choi D, Park JM (2006). Suppression of *CaCYP1*, a novel cytochrome P450 gene, compromises the basal pathogen defense response of pepper plants.. Biochemical and Biophysical Research Communications.

[70] Lecourieux D, Raneva R, Pugin A (2006). Calcium in plant defence-signalling pathways.. New Phytologist.

[71] Wang Y, Liu JH (2012). Exogenous treatment with salicylic acid attenuates occurrence of citrus canker in susceptible navel orange (*Citrus sinensis* Osbeck).. Journal of Plant Physiology.

[72] Breitling R, Armengaud P, Amtmann A, Herzyk P (2004). Rank Products: A simple, yet powerful, new method to detect differentially regulated genes in replicated microarray experiments.. FEBS Letters.

[73] Benjamini Y, Hochberg Y (1995). Controlling the false discovery rate: a practical and powerful approach to multiple testing.. Journal of the Royal Statistical Society: Series B (Statistical Methodology).

[74] Sun Q, Zybailov B, Majeran W, Friso G, Dominic P (2009). PPDB, the plant proteomics database at Cornell.. Nucleic Acids Research.

[75] Benjamini Y, Yekutieli D (2001). The control of the false discovery rate in multiple testing under dependency.. Annals of Statistics.

