# Supplementary figures and images for: Different Transcriptional Response to Xanthomonas citri subsp. citri between Kumquat and Sweet Orange with Contrasting Canker Tolerance
Source: PLoS One. 2012 Jul 26;7(7):e41790. doi: 10.1371/journal.pone.0041790 (PMC3406098; doi:10.1371/journal.pone.0041790)

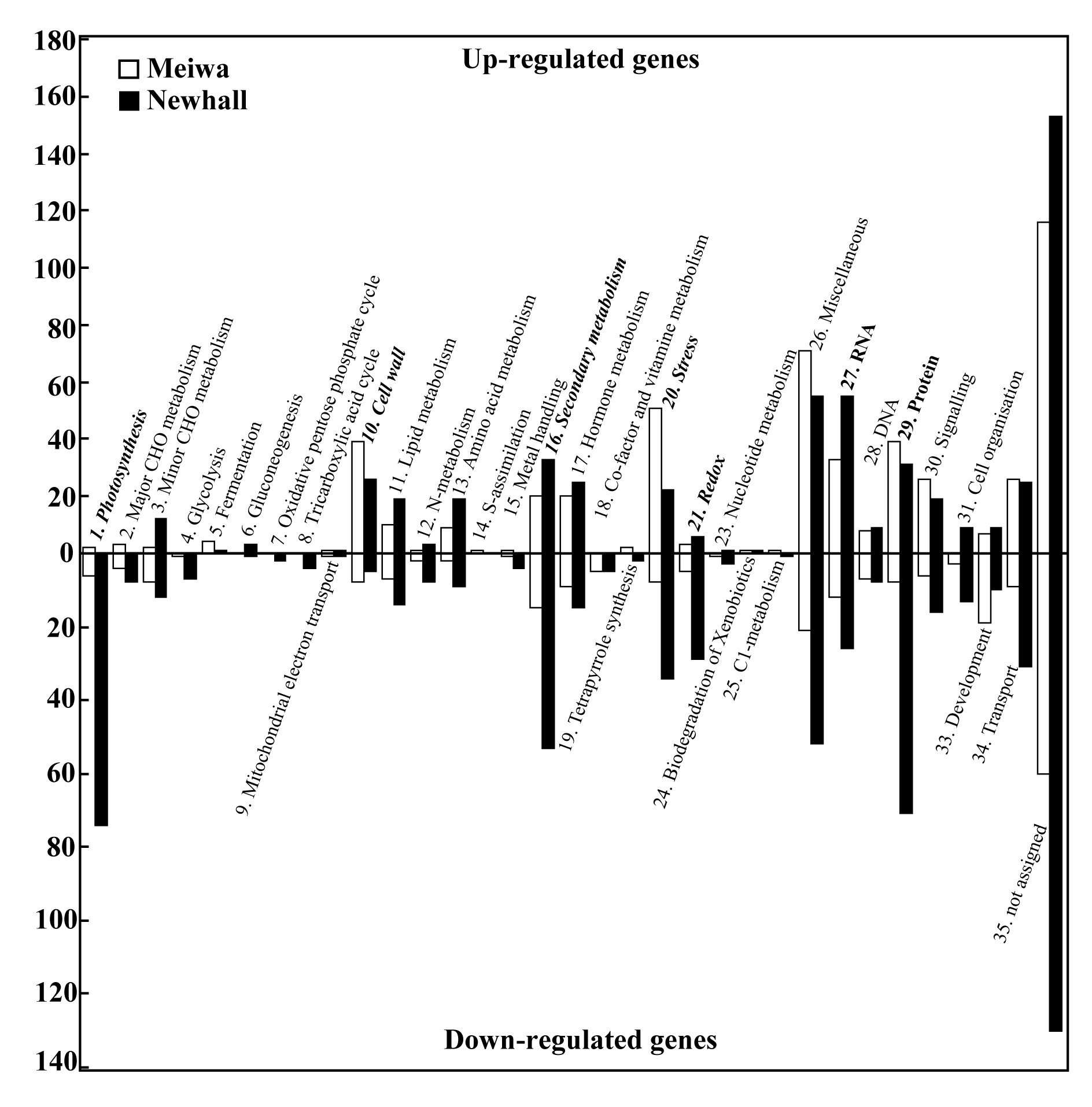

Supplement: Figure S1 — MapManbin classification of differentially expressed genes in ‘Meiwa’ and ‘Newhall’. (TIF) [file pone.0041790.s001.tif]
